# Fasting during cancer treatment: a systematic review

**DOI:** 10.1007/s11136-022-03300-1

**Published:** 2022-11-28

**Authors:** U. Drexler, J. Dörfler, J. von Grundherr, N. Erickson, J. Hübner

**Affiliations:** 1 Klinik für Innere Medizin II, Hämatologie und Internistische Onkologie, Universitätsklinikum Jena, Am Klinikum 1, 07747 Jena, Germany; 2 Hubertus Wald Universitäres Cancer Center Hamburg (UCCH), Martinistraße 54, 20246 Hamburg, Germany; 3 Comprehensive Cancer Center (CCC Munich LMU), LMU University Hospital Munich, Marchioninistraße 15, 81377 Munich, Germany

**Keywords:** Cancer, Nutrition, Diet, Fasting, Side effects, Chemotherapy

## Abstract

**Background:**

Clinical data on the modern topic fasting among cancer patients are rare. This review aimed to summarise published clinical data on fasting and its effects on patients undergoing chemotherapy and therefore to give some directions in advising patients with the desire to fast.

**Method:**

A systematic search was conducted searching five electronic databases (Embase, Cochrane, PsychInfo, CINAHL and Medline) to find studies concerning the use, effectiveness and potential harm of fasting during therapy on cancer patients. The main endpoints were quality of life, side effects and toxicities of the fasting intervention.

**Results:**

The search results totaled 3983 hits. After systematic sorting according to standardised pre-defined criteria, nine publications which covered eight studies with 379 patients were included in this systematic review. The majority of the patients included were diagnosed with breast- and gynaecological cancers. Fasting duration and timepoints ranged significantly (24–140 h before, and on the day of, chemotherapy to 56 h after chemotherapy). In one study patients were fasting before cancer surgery. The studies were mostly low to moderate quality and reported heterogeneous results. Overall, the studies were insufficiently powered to detect significant effects on the predefined endpoints.

**Conclusion:**

Fasting for short periods does not have any beneficial effect on the quality of life of cancer patients during treatment. Evidence on fasting regimes reducing side effects and toxicities of chemotherapy is missing. In contrast, as the negative effects of unintentional weight loss are known to impact clinical outcomes severely, fasting is not indicated in this context.

## Introduction

Nutrition plays a central role in cancer care. Malnutrition, as well as obesity, is associated with a much lower prognosis for most cancer entities [1–3]. Recently, the fasting concept has become popular among scientists and the general population. In particular, fasting reputedly slows down ageing, improves overall health [4–6] and supposedly even prevents illnesses such as cancer, hypertension, or diabetes [7–9]. Preclinical studies in mice found an increased resistance to oxidative stress and an extended lifespan [10]. Bagherniya et al. tested neuroblastoma-bearing mice consuming only water for 48 h prior to treatment with etoposide and concluded that this intervention potentially protects against toxicity of the chemotherapy [11]. Proposed mechanisms for this effect include an induction of autophagocytosis of cancer cells and/or the idea that fasting may also protect healthy cells in cancer patients against the stress induced by chemotherapy [12]. A meta-analysis from studies in murine models showed that caloric restriction is effective against cancer [13] as it sensitises some types of cancer cells to chemotherapy [14]. As these preclinical studies have shown promising results, there is high interest in transferring these findings to humans. One of the first to observe the effect of fasting on cancer treatment in 10 humans were Safdie et al. in 2009 [15]. In Germany, a randomized controlled trial was done by Bauersfeld et al. [16] at the Charité-University in Berlin, which has a very big impact on the scientific research and clinical practice, due to being one of the leading hospitals in clinical research in Germany. Currently, it is not clear whether there is also high-quality clinical evidence for fasting during treatment for cancer in humans. Most of the published data on this topic deal with preventing cancer but not with a treatment-associated intervention on patients undergoing chemotherapy or surgery. To our knowledge, so far no systematic review on this topic exists. Therefore, the aim of this systematic review was to summarise the clinical evidence of fasting before chemotherapy treatment on patients’ quality of life and on chemotherapy-related toxicities. As fasting can be done by almost everyone without the need of, for example, medical prescription or supervision, the disadvantages and potential harms were investigated. Moreover, the side effects and adverse events in studies on fasting were assessed.

## Methods

### Inclusion and exclusion criteria

The Patient/Population-Intervention-Comparison-Outcome (PICO) model served as a basis for the definition of inclusion and exclusion criteria [17]. All clinical study types (systematic reviews, randomised controlled trials, controlled trials, one-armed studies, and case series) were included if they reported patient-relevant outcomes after treatment of adult cancer patients with any intervention containing dietary interventions like fasting or caloric restriction (defined as less than 400 kcal/day). All cancer entities were included. Criteria for rejecting studies were animal studies, primary prevention, grey literature, other publication type than primary investigation/report (e.g. comments, letters, abstracts) or precancerous conditions. Additionally, studies were excluded if they reported no patient centered outcomes (for example only laboratory data). The search was restricted to publications in English or German. A short overview of inclusion and exclusion criteria can be found in Table 1.

**Table 1:** Inclusion & exclusion criteria

Inclusion criteria	Exclusion criteria
Cancer patients (all types) Intervention: fasting, caloric restriction patient-reported outcomes, adverse events randomized controlled trial, controlled trial, systematic review, one-armed studies, case series	Healthy patients, patients with precancerous lesions primary prevention, preclinical study (in vivo, in vitro) multimodal interventions without separate evaluation laboratory data grey literature (abstracts, letters, ongoing trials) Reviews without systematic research

### Study selection

The systematic research was conducted using five databases [Medline (Ovid), CINAHL (EBSCO), EMBASE (Ovid), Cochrane CENTRAL and PsycINFO (EBSCO)] in May 2021. A combination of MeshTerms, keywords, and text words in different spellings connected to cancer and fasting was consistently applied for each database search (Table 2). The search string was considered highly sensitive, as it was not restricted by filters for study or publication type. After importing the search results into EndNote X9 all duplicates were removed and a title- abstract- screening was carried out by two independent reviewers (UD, JD). In case of disagreement consensus was made by discussion or consulting a third reviewer (JH). When title and abstract did not include sufficient information for screening purposes, a full-text copy was retrieved. Additionally, the bibliography lists cited in all retrieved articles were searched for relevant studies. Finally, the full-text version of each article was screened independently by both reviewers.

**Table 2:** Database search

Database	Searchstring
Ovid Medline	1 exp Fasting/ OR ((Exp diet therapy/ OR diet.mp.) AND (fasting).mp.)
	2 exp neoplasms/ or neoplasm$.mp or cancer$.mp. or tumo?r$.mp. or malignan$.mp. or oncolog$.mp. or carcinom$.mp. or leuk?emia.mp. or lymphom$.mp. or sarcom$.mp
	3 1 AND 2
	4 limit 3 to english or limit 3 to german
	5 (4 and humans/) or (4 not animals/)
	6 ((((comprehensive* or integrative or systematic*) adj3 (bibliographic* or review* or literature)) or (meta-analy* or metaanaly* or "research synthesis" or ((information or data) adj3 synthesis) or (data adj2 extract*))).ti,ab. or (cinahl or (cochrane adj3 trial*) or embase or medline or psyclit or (psycinfo not "psycinfo database") or pubmed or scopus or "sociological abstracts" or "web of science" or central).ab. or ("cochrane database of systematic reviews" or evidence report technology assessment or evidence report technology assessment summary).jn. or Evidence Report: Technology Assessment*.jn. or (network adj1 analy*).ti,ab.) or (((review adj5 (rationale or evidence)).ti,ab. and review.pt.) or meta-analysis as topic/ or Meta-Analysis.pt.)
	7 Randomi?ed controlled trial?.pt. or controlled clinical trial?.pt. or randomi?ed.ti,ab.or placebo.ti,ab. or drug therapy.sh. or randomly.ti,ab. or trial?.ti,ab. or group?.ti,ab
	8 5 AND (6 OR 7)
	9 5 NOT 8
Ovid Embase	1 exp diet restriction OR ((exp diet / OR exp diet therapy/ OR diet.mp.) AND (fasting).mp.)
	2 exp neoplasm/ or neoplasm$.mp or cancer$.mp. or tumo?r$.mp. or malignan$.mp. or oncolog$.mp. or carcinom$.mp. or leuk?emia.mp. or lymphom$.mp. or sarcom$.mp
	3 1 AND 2
	4 limit 3 to english or limit 3 to german
	5 (4 and humans/) or (4 not animals/)
	6 ((((comprehensive* or integrative or systematic*) adj3 (bibliographic* or review* or literature)) or (meta-analy* or metaanaly* or "research synthesis" or ((information or data) adj3 synthesis) or (data adj2 extract*))).ti,ab. or (cinahl or (cochrane adj3 trial*) or embase or medline or psyclit or (psycinfo not "psycinfo database") or pubmed or scopus or "sociological abstracts" or "web of science" or central).ab. or ("cochrane database of systematic reviews" or evidence report technology assessment or evidence report technology assessment summary).jn. or Evidence Report: Technology Assessment*.jn. or (network adj1 analy*).ti,ab.) or (exp Meta Analysis/ or ((data extraction.ab. or selection criteria.ab.) and review.pt.))
	7 crossover procedure/ or double blind procedure/ or randomized controlled trial/ or single blind procedure/ or (random$ or factorial$ or crossover$ or (cross adj1 over$) or placebo$ or (doubl$ adj1 blind$) or (singl$ adj1 blind$) or assign$ or allocat$ or volunteer$).ti,ab,de
	8 5 AND (6 OR 7)
	9 5 NOT 8
Cochrane	#1 [mh fasting] or ([mh “diet therapy”] OR diet) AND fasting
	#2 [mh neoplasms] or neoplasm* or cancer? or tum*r? or malignan* or oncolog* or carcinom* or leuk*mia or lymphoma? or sarcoma?
	#3 #1 AND #2
Ebsco—PsychINFO	S1 (DE “Diet” OR TX diet) AND TX (fasting)
	S2 ((DE "Neoplasms" OR DE "Benign Neoplasms" OR DE "Breast Neoplasms" OR DE "Endocrine Neoplasms" OR DE "Leukemias" OR DE "Melanoma" OR DE "Metastasis" OR DE "Nervous System Neoplasms" OR DE "Terminal Cancer") OR (TX neoplasm* OR TX cancer OR TX tumo#r OR TX malignan* OR DE „oncology “ OR TX oncolog* OR TX carcinom* OR TX leuk#emia OR TX lymphoma OR TX sarcoma))
	S3 (LA German OR LA English)
	S4 S1 AND S2 AND S3
	S5 ((comprehensive* OR integrative OR systematic*) N3 (bibliographic* OR review* OR literature)) OR (meta-analy* or metaanaly* or "research synthesis" OR ((information OR data) N3 synthesis) OR (data N2 extract*)) OR ((review N5 (rationale OR evidence)) AND DE "Literature Review") OR (AB(cinahl OR (cochrane N3 trial*) OR embase OR medline OR psyclit OR pubmed OR scopus OR "sociological abstracts" OR "web of science" OR central)) OR DE "Meta Analysis" OR (network N1 analy*)
	S6 DE "Treatment Effectiveness Evaluation" OR DE "Treatment Outcomes" OR DE "Psychotherapeutic Outcomes" OR DE "Placebo" or DE "Followup Studies" OR placebo* OR random* OR "comparative stud*" OR (clinical N3 trial*) OR (research N3 design) OR (evaluat* N3 stud*) OR (prospectiv* N3 stud*) OR ((singl* OR doubl* OR trebl* OR tripl*) N3 (blind* OR mask*)
	S7 S4 AND (S5 OR S6)
	S8 S4 NOT S7
Ebsco- CINAHL	S1 MH “Fasting” OR ((MH “Diet + ” OR TX diet) AND TX (fasting)
	S2 (MH "Neoplasms + " OR TX neoplasm* OR TX cancer OR TX tumo#r OR TX malignan* OR TX oncolog* OR TX carcinom* OR TX leuk#emia OR TX lymphoma OR TX sarcoma)
	S3 (LA German OR LA English)
	S4 S1 AND S2 AND S3
	S5 (TI (systematic* n3 review*)) or (AB (systematic* n3 review*)) or (TI (systematic* n3 bibliographic*)) or (AB (systematic* n3 bibliographic*)) or (TI (systematic* n3 literature)) or (AB (systematic* n3 literature)) or (TI (comprehensive* n3 literature)) or (AB (comprehensive* n3 literature)) or (TI (comprehensive* n3 bibliographic*)) or (AB (comprehensive* n3 bibliographic*)) or (TI (integrative n3 review)) or (AB (integrative n3 review)) or (JN “Cochrane Database of Systematic Reviews”) or (TI (information n2 synthesis)) or (TI (data n2 synthesis)) or (AB (information n2 synthesis)) or (AB (data n2 synthesis)) or (TI (data n2 extract*)) or (AB (data n2 extract*)) or (TI (medline or pubmed or psyclit or cinahl or (psycinfo not “psycinfo database”) or “web of science” or scopus or embase)) or (AB (medline or pubmed or psyclit or cinahl or (psycinfo not “psycinfo database”) or “web of science” or scopus or embase or central)) or (MH “Systematic Review”) or (MH “Meta Analysis”) or (TI (meta-analy* or metaanaly*)) or (AB (meta-analy* or metaanaly*)) or network n1 analy*
	S6 (MH "Clinical Trials + ") or PT Clinical trial or TX clinic* n1 trial* or TX ( (singl* n1 blind*) or (singl* n1 mask*)) or TX ((doubl* n1 blind*) or (doubl* n1 mask*)) or TX ( (tripl* n1 blind*) or (tripl* n1 mask*)) or TX ((trebl* n1 blind*) or (trebl* n1 mask*)) or TX randomi* control* trial* or (MH "Random Assignment") or TX random*
	allocat* or TX placebo* or MH "Placebos") or MH "Quantitative Studies") or TX allocat* random*
	S7 S4 AND (S5 OR S6)
	S8 S4 NOT S7

### Assessment of risk of bias and methodological quality

Predefined characteristics were used to assess the risk of bias and methodological quality by two independent reviewers (UD, JD). In case of disagreement, a third reviewer was consulted (JH) and consensus was reached. The interrater reliability was assessed using Cohen’s kappa (0.79), which can be interpreted as close to very good [18].

Risk of bias was analysed with the SIGN-Checklist for controlled trials Version 2.0 and AMSTAR-2 instrument for systematic reviews or meta-analyses [19] and the Institute of Health Economics (IHE) Quality Appraisal Checklist for Case Series Studies [20]. Additionally, blinding of researchers, blinding of outcome assessment and comparability of groups before treatment, not only in terms of demographic variables but also with relation to outcomes were examined.

The included studies were rated with the Jadad Scale [21]. Additional criteria concerning methodology were population size, power analysis calculation, handling of missing data, drop-out rate and reasons (report of drop-out reasons, intention-to-treat-analysis), adequacy of statistical tests (e.g. control of premises or multiple testing) and finally, full outcome reporting (report of all assessed outcomes with specification of effect estimate and *p* value).

### Data extraction

Data extraction was performed by one reviewer (UD) and controlled by two independent reviewers (JD, JH). As a template for data extraction, the evidence tables from the national Guideline on Complementary and Alternative Medicine in Oncological Patients of the German Guideline Program in Oncology [22] were used. These tables include study type, study population, interventions, endpoints, and effect estimates (Table 3), methodical limitations and grade of evidence (Table 5).

**Table 3:** Overview of included studies

Reference	Study type	*n*/drop-out	Intervention/duration	Endpoints	Outcomes
Bauersfeld [16]	RCT	*n* = 50 Drop-Out = 16	Arm A: Fasting (36 h before, 24 h after) for 1st 3 chemotherapy cycles (T1) & normocaloric diet for 2nd 3 chemotherapy cycles (T2)	T0: Baseline day 0 cycle 1	
				T1: Cycle 1–3 (Group A fasting, Group B normocaloric)	
				T2: Cycle 4–6 (Group A normocaloric, Group B fasting)	
			Arm B: Normocaloric diet for 1st 3 chemotherapy cycles (T1) & fasting (36 h before, 24 h after) for 2nd 3 chemotherapy cycles (T2)	1. Quality of Life FACT-G scale	1: Significant increase in QOL in Arm A during T1, no significant changing in Arm B, no group comparison was conducted
					QOL: mean (SD): Arm A pretest: 71.1 (18.7), posttest: 61.9 (18.5), *p* = 0.04, Arm B pretest: 76 (17.2), posttest: 74 (17.6), *p* > 0.05
				2. Quality of Life FACIT-F scale	2: Significant increase of QOL in Arm A during T1, no significant changing in Arm B, no group comparison was conducted
					Arm A pretest: 33.9 (13.4), posttest: 24.8 (13.7), *p* = 0.006, Arm B pretest: 33.4 (14), posttest: 31.7 (12.6), *p* > 0.05
				3. Quality of Life FACIT-F TOI scale	3: Significant increase in QOL in Arm A during T1, no significant changing in Arm B, no group comparison was conducted
			Duration: Fasting for 60 h during each chemotherapy cycle		Arm A pretest: 66 (25), posttest: 49.8 (26.4), *p* = 0.009, Arm B pretest: 68 (25.2), posttest: 63.8 (23.9), *p* > 0.05
				4. Quality of Life FACIT-F Total	4: Significant increase in QOL in Arm A during T1, no significant changing in Arm B, no group comparison was conducted
					Arm A Mean Difference: 18.3, *p* = 0.013, Arm B Mean Difference: 4.2, *p* > 0.05
				5. Bodyweight/BMI	5: No significant changing in bodyweight from T1 to T2 in Arm A, no significant changing in bodyweight from T1 to T2 in Arm B, no group comparison was conducted
					Bodyweight in kg: Arm A pretest: 73, posttest: 72.3, *p* > 0.3, Arm B pretest: 67.9, posttest: 68.5, *p* > 0.3
De Groot [28]	RCT	*n* = 13	Arm A: Fasting (24 h before, 24 h after) chemotherapy for 6 cycles	1. Chemotherapy-induced side effects Grade I/II (fatigue, infection, mucositis, neuropathy, diarrhea, dizziness, nausea, eye complaints, constipation)	1: No significant differences between both groups, *p* not stated
		Drop-Out = 2	Arm B: Eating according to guidelines for healthy nutrition	2. Chemotherapy-induced side effects Grade III/IV (neutropenic fever, fatigue, infection)	2: No significant differences between both groups, *p* not stated
			Duration: Fasting for 48 h for 6 cycles of chemotherapy, 3 weeks each (18 weeks in total)		
De Groot [23]	RCT	*n* = 131	Arm A: Fasting mimicking diet 3 days prior to and during the day of each cycle of chemotherapy	1. Chemotherapy-related toxicities CTCAE (Grade III/IV)	1: No significant difference between Arm A & Arm B
		Drop-Out = 50			Arm A: 31 patients (47.7%), Arm B: 36 patients (56.3%), *p* > 0.05
				2. pathological complete response	2: No significant difference between Arm A & Arm B
			Arm B: Consuming regular diet		(%): Arm A: 10.8, Arm B: 12.7, *p* > 0.05
			Duration: 6–8 cycles of chemotherapy	3. radiological response RECIST scala	3: Significant higher radiological response in Arm A
					(%): Arm A: 47, Arm B: 38, *p* = 0.039
				4. pathological response Miller & Payne Score 4/5	4: No significant difference between Arm A & Arm B
					(%): Arm A: 20, Arm B: 14, *p* > 0.05 but compliant patients in Arm A significant higher pathological response
					(%): Arm A: 10, Arm B: 12, *p* = 0.016
				5. Quality of Life EORTC QLQ-C30	5: No significant difference between Arm A & Arm B, *p* > 0.05
				6. Psychological distress thermometer	6: No significant difference between Arm A & Arm B, *p* > 0.05
				7. DNA Damage	7: Significant less DNA Damage in Arm A in CD45/CD3 + T-Lymphocytes
					(%): Arm A: 16, Arm B: 11, *p* = 0.045
Lugtenberg [24] same RCT as De Groot [23] with different Endpoints	RCT	*n* = 131	Arm A: Fasting mimicking diet 3 days prior to and during the day of each cycle of chemotherapy	1. Quality of Life Baseline (EORTC-QLQ-C30/EORTC BR23/BIPQ/Distress Thermometer)	1: No significant difference between Arm A & Arm B, *p* > 0.05
		Drop-out = 50		2. Quality of Life Halfway Therapy (EORTC-QLQ-C30/EORTC B23/Distress Thermometer)	2: No significant difference between Arm A & Arm B, *p* > 0.05
			Arm B: Consuming regular diet		
			Duration: 6–8 cycles of chemotherapy	3. Quality of Life before last cycle of chemotherapy (EORTC-QLQ-C30/EORTC b23/ Distress Thermometer)	3: No significant difference between Arm A & Arm B, *p* > 0.05
				4. Quality of Life 6-month-Follow-Up (EORTC-QLQ-C30/EORTC B23/ Distress Thermometer)	4: No significant difference between Arm A & Arm B, *p* > 0.05
				5. Global Health Status (EORTC-Qol-C30)	5: During Treatment significant reduction in Global Health in each Arm returning to Baseline during Follow-Up, *p* < 0.01
				6. Illness perception (BIPQ)	6: No significant difference between Arm A & Arm B, but Arm A seems to understand toxicities better (*p* = 0.01) and has significant less worries (*p* < 0.01)
Lende [27]	RCT	*n* = 80	Arm A: Fasting before breast cancer surgery (standard fasting procedure)	1. Quality of life patient-recorded outcome measures (nausea, pain, mobilization, dizziness, insecurity, bleeding)	1: Significant more reports of mild & moderate pain 7 days post-OP in Arm A, *p* < 0.001
					in post-hoc analysis significant on day 5 ( Arm A: 47%, Arm B: 28%, *p* = 0.038) & day 6 (Arm A: 50%, Arm B: 28%, *p* < 0.001)
		Drop-Out = 19	Arm B: 2 × Pre-operative carbohydrates (400 ml pre-OP Nutricia 18 h before & 2-3 h before surgery)	2. Relapse-free survival (surgery until diagnosis of relapse) in whole cohort	2: Significant higher chance of relapse-free survival in Arm A
					Event at risk (% survival), *p *value: Arm A: 2/35 (94), Arm B: 6/26 (77), *p* = 0.049
				3. Relapse-free survival in ER + patients (endocrine resistant tumours to Insulin/IGF1-pathway)	3: Significant higher chance of relapse-free survival in Arm A
					Arm A: 1/29 (97), Arm B: 6/21 (71), *p* = 0.012
			Duration: standard fasting procedure, exact length not stated in study	4. Breast cancer-specific survival (surgery until death due to breast cancer) in whole cohort	4: No significant differences between the two groups
					Arm A: 1/35 (97), Arm B: 4/26 (85), *p* = 0.08
				5. Breast cancer-specific survival in ER + patients	5: Significant higher chance of breast cancer-specific survival in Arm A
					Arm A: 0/29 (100), Arm B: 4/21 (81), *p* = 0.015
				6. Overall survival (surgery until death from any cause)	6: no report, Cox model was considered too unstable
Riedinger [25]	RCT	*n* = 24	Arm A: Fasting 24 h before and 24 h following chemotherapy	1. Quality of life NCCN FACT FOSI-18 score	1: No significant difference between Arm A & Arm B
					Mean, *p* value: Arm A: 58.38, Arm B: 57.1, *p* = 0.71
					but Arm A significant higher score during one cycle compared to baseline compared to Arm B
		Drop-Out = 4	Arm B: Eating a normocaloric diet		Score, *p *value: Arm A: + 5.1, Arm B: + 0.22, *p* = 0.015
			Duration: Chemotherapy cycles 3-week intervals fot a minimum of 6 cycles, in total 120 cycles	2. Chemotherapy-related side effects (a: CTCAE toxicity Grade 3 or 4, b: Anemia Grade 2, c: Thrombocytopenia Grade 2, d: Neutropenia Grade 2 / 3)	2a: No significant difference between Arm A & Arm B
					Arm A 0 patients, Arm B 2 patients, *p* > 0.05
					2b: No significant difference between Arm A & Arm B
					Arm A 1 patient, Arm B 3 patients, *p* > 0.05
					2c: No significant difference between Arm A & Arm B
					Arm A 0 patients, Arm B 1 patient, *p* > 0.05
					2d: No significant difference between Arm A & Arm B
					Arm A: 4 patients / 0 patients, Arm B: 2 patients / 1 patient, *p* > 0.05
				3. Hematologic parameters (a: Hemoglobin, b: Neutrophil count, c: Platelet count, d: Serum glucose)	3a: No significant difference between Arm A & Arm B
					Mean (SD), *p* value: Arm A: 10.8 (1.3), Arm B: 10.9 (1.5), *p* > 0.05
					3b: No significant difference between Arm A & Arm B
					Mean (SD), *p *value: Arm A: 4.7 (3.3), Arm B: 4 (2.2), *p* > 0.05
					3c: Significant higher Platelet count in Arm A
					Mean (SD), *p *value: Arm A: 246.5 (96.5), Arm B: 203.5 (82.5), *p* = 0.009
					3d: Significant less Serum glucose in Arm A
					Mean (SD), *p* value: Arm A: 97.2 (17.8), Arm B: 120 (25.6), *p* < 0.001
				4. Change in bodyweight	4: No significant difference between Arm A & Arm B
					Mean (SD), *p *value: Arm A: 0.84 (2.33), Arm B: 1.1 (3.55), *p* > 0.05
Safdie [15]	Case report	*n* = 10	Fasting before/after chemotherapy	Side effects of fasting	1: loss of 7 pounds (recovered after treatment), mild fatigue, dry mouth, hiccups
					2: loss of 7 pounds (regained 4), less severe side effects compared to cycles with calories, mild diarrhea, abdominal cramps
					3: lower side effects compared to previous cycles with calories, PSA reduced from 34 to 6
					4: loss of 6 pounds (recovered), mild fatigue, weakness, acute toxic side effects reduced, quick regain of strength
			Duration: fasting for 48–140 h before and/or 5–56 h after		5: reduced side effects
					6: faster recovery of blood cell counts
					7: lightheadedness, drop in blood pressure, neuropathy
					8: mild weakness, short-term memory impairment
					9: mild weakness, alopecia
Zorn [26]	Controlled Cross Over Study	*n* = 51	Double-Intervention Fasting in combination with ketogenic diet	1. Chemotherapy-related toxicities CTCAE Grade I/II (Infection, Fatigue, Insomnia, Headaches; Dizziness, Depression, Nausea, Vomiting, Diarrhea, Obstipation, Stomach pains, Reduced appetite, Hunger, Stomatitis, Esophagitis, Neuroses, Arthralgia, Pain, Dyspnea, Oedemas)	1. No significant difference between the groups except for Stomatitis
					Stomatitis Mean (%), *p* value: Normocaloric Cycles: 15 (25.9); Fasted Cycles: 3 (5.5), *p* = 0.013
		Drop-Out = 21	Arm A: 2–3 chemotherapy cycles modified short-term fast 3 days prior & 1 day after chemotherapy, followed by 2–3 chemotherapy cycles on a normocaloric diet	2. Chemotherapy-related toxicities 1 week after chemotherapy, patient-reported (Reduced appetite, Hunger, Nauseam Vomiting, Stomach pains, Diarrhea, Obstipation, Fever, Headaches, Insomnia, Fatigue, Dizziness, Weakness, Exhaution, frequency & score of total toxicities)	2: No significant difference between the groups except for headaches, weakness & total toxicities
					Headaches Mean (SD), *p* value: Fasted Cycles: 1.18 (2.06), Normocaloric Cycles: 2.74 (4.3), *p* = 0.002
					Weakness Mean (SD), *p *value: Fasted Cycles: 2.84 (4.25), Normocaloric Cycles: 7.11 (5.4), *p* = 0.024
					Total Toxicities Mean (SD), *p *value: Fasted Cycles: 47.52 (33.21), Normocaloric Cycles: 56.36 (32.14), *p* = 0.023
			Arm B: 2–3 chemotherapy cycles normocaloric diet followed by 2–3 modified short-term fast	3. Blood parameters (MCV, MCH, Erythrocytes, Sodium, fT3/fT4)	3: No significant difference between the groups except for MCV, MCH, sodium & fT3/fT4
			Arm C: 2–3 cycles modified short-term fast with a normocaloric ketogenig diet 6 days prior to fasting, followed by 2–3 cycles normocaloric diet		MCV Mean (SD), *p *value: Fasted Cycles: 84.1 (3.4), Normocaloric Cycles: 85.7 (4), *p* < 0.001
					MCH Mean (SD), *p* value: Fasted Cycles: 29.5 (1.6), Normocaloric Cycles: 29.9 (1.5), *p* = 0.004
					Sodium Mean (SD), *p* value: Fasted Cycles: 137.9 (3.3), Normocaloric Cycles: 139.2 (2.2), *p* = 0.007
					fT3 Mean (SD), *p* value: Fasted Cycles: 3.75 (0.76), Normocaloric Cycles: 4.21 (0.81), *p* < 0.001
					fT4 Mean (SD), *p* value: Fasted Cycles: 16.4 (2.9), Normocaloric Cycles: 15.5 (2.7), *p* = 0.028
			Arm D: 2–3 chemotherapy cycles normocaloric diet followed by 2–3 cycles modified short-term fast with a normocaloric ketogenic diet 6 days prior to fasting		
				4. Quality of Life, CIPN, Fatigue	4: No significant difference between the groups, *p* not stated
				5. Number of days postponing chemotherapy due to toxicities	5: Significant less postponements during Fasted Cycles
			Duration: 56 cycles fasted, 62 cycles normocaloric diet		Mean (SD), *p *value: − 0.8 (0.37), *p* = 0.034
Dorff [29]	Cohort study	*n* = 13	Arm A: Fasting 24 h before platinum-based chemotherapy	1. Chemotherapy-related toxicities (a: fatigue, b: alopecia, c: nausea, d: vomiting, e: constipation, f: diarrhea)	1a: Arm A: 6/6 (100%), Arm B: 5/7 (71%), Arm C: 6/7 (86%)
					1b: Arm A: 6/6 (100%), Arm B: 5/7 (71%), Arm C: 7/7 (100%)
		Drop-out = 3	Arm B: Fasting 48 h before p.b.-chemotherapy		1c: Significant more patients nauseous in Arm A > Arm B > Arm C
			Arm C: Fasting 48 h before & 24 h after p.b.-chemotherapy		Arm A: 6/6 (100%), Arm B: 6/7 (86%), Arm C: 3/7 (43%), *p* = 0.019, test for trend
					1d: Significant more patients vomited in Arm A > Arm B > Arm C
			Duration: 24 h/48 h/72 h of fasting		Arm A: 5/6 (83%), Arm B: 3/7 (43%), Arm C: 0/7 (0%), *p* = 0.003, test for trend
					1e: Arm A: 3/6 (50%), Arm B: 2/7 (28%), Arm C: 3/7 (43%)
					1f: Arm A: 2/6 (33%), Arm B: 1/7 (14%), Arm C 4/7 (57%)

*SD* Standardized difference; *QoL* Quality of Life; *FACT- B* Functional Assessment of Cancer Therapy in patients with Breast cancer; *FACT-G* Functional Assessment of Cancer Therapy –General; *FACT/GOG-NTX* Functional Assessment of Cancer Therapy/Gynecologic Oncology Group-Neurotoxicity

## Results

**Fig. 1:**
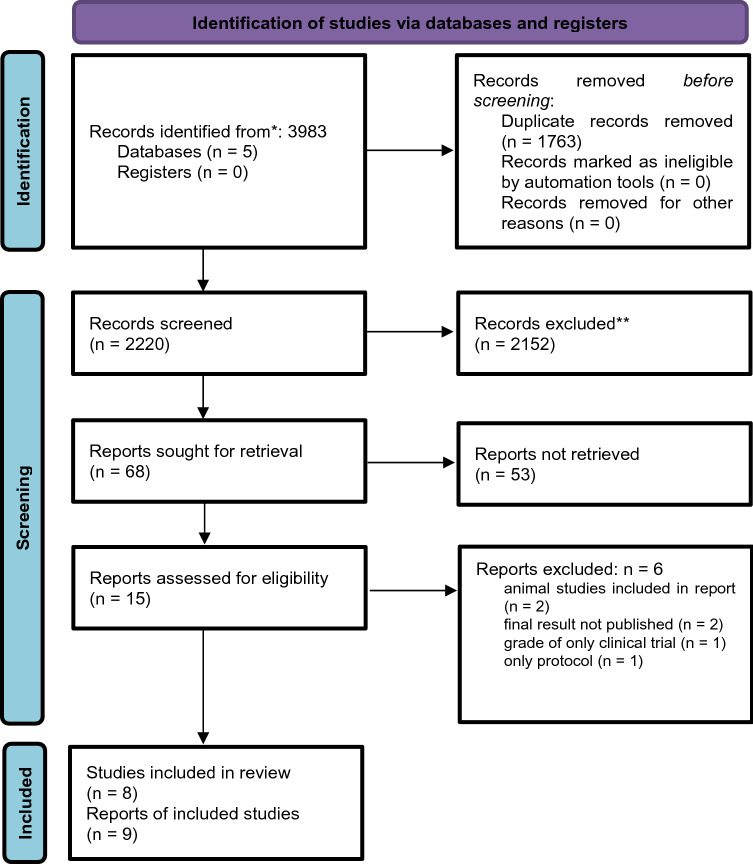
Flowchart

The systematic research revealed 3983 results. No studies were added by hand search. After duplicates were removed 2220 studies remained. After screening the titles and abstracts, 68 studies underwent a comprehensive review; 53 of these studies were excluded due to various reasons (no relation to cancer or fasting, only assessing the risk of getting cancer, only reporting medication/diagnosis/prevention, and 6 were excluded after eligibility assessment). In the end, nine publications were analysed in this review, including six randomised controlled trials (RCTs), one controlled cross-over study, one cohort study and one case report. In total, nine publications reported data from eight studies (Table 3). The study which was reported in two articles focused on different outcomes [23, 24]. Detailed characterisation of the included studies and excluded studies is provided (Tables 3 and 4, respectively). A flow diagram is included (Fig. 1).

**Table 4:** Excluded studies

Author	Year	Title	Exclusion criteria
Lv	2014	Roles of caloric restriction, ketogenic diet and intermittent fasting during initiation, progression and metastasis of cancer in animal models: a systematic review and meta-analysis	Animal studies included
Sun	2017	Effect of fasting therapy in chemotherapy-protection and tumorsuppression: A systematic review	Animal studies included
Bertz	2018	MOFAX Impact of short-term modified fasting and the combination with a fasting supportive diet during chemotherapy on the incidence and severity of chemotherapy-induced toxicities in cancer patients-a randomised controlled cross-over pilot study	Final results not published, single parts of these interventions were not analyzed separately
Dorff	2018	A randomized phase II clinical trial of a fasting-mimic diet prior to chemotherapy to evaluate the impact on toxicity and efficacy	Grade of evidence/publication, only clinical trial
Shingler	2019	A feasibility randomised controlled trial of short-term fasting prior to CAPOX chemotherapy for stage 2/3 colorectal cancer: sWiFT protocol	Publication, only protocol
De Braud	2018	Safety and metabolic effects of cyclic fasting mimicking diet (FMD) in cancer patients	Final results not published, single parts of these interventions were not analyzed separately

In total, 379 patients were included in the nine studies of this review. Due to 71 drop outs, 308 of these patients were analysed according to intention to treat principle. Patient age ranged from 27 to 78 years. The majority of study participants were females (373 females vs. six males). Fasting periods varied significantly between studies. An exact indication of the duration can be found in “Intervention” column of Table 3.

### Excluded studies

Two systematic reviews were excluded due to inclusion of animal studies; four trials with multiple interventions in addition to fasting were also excluded as the effects of the single parts of these interventions were not analysed separately, so it was not possible to estimate whether the reported effects were caused by the fasting therapy or by another treatment. Excluded studies with detailed exclusion reasons can be found in Table 4.

### Risk of bias in included studies

Methodical quality was assessed using the SIGN-Checklist for controlled trials. All the included studies have low to moderate quality (Table 5).

**Table 5:** Bias assessement

Reference	Study type	Standardized rating of risk of bias	Additional comments on methodology	Evidence Level (Oxford)
Bauersfeld [16]	RCT	SIGN	PRO: poweranalysis was conducted; structured explanation of statistical analysis; use of different scales for validation of outcome	1b
		Positive: 5		
		Uncertain: 2		
		Negative: 1	CONTRA:	
		Overall quality: Acceptable	Patients & blinding: very small sample size (*n*=34) analysed; patients not blinded; not placebo-controlled methods: significant differences between the groups not statistically documented (confusing report in text, partially not proven with numbers), multiple testing	
De Groot [28]	RCT	SIGN	CONTRA:	2b
		Positive: 4	Patients & blinding: very small sample size (*n* = 11); patients not blinded	
		Uncertain: 2		
		Negative: 1	Methods: no poweranalysis was conducted; randomization not specified	
		Overall quality: Acceptable		
De Groot [23]	RCT	SIGN	PRO: poweranalysis was conducted; structured explanation of randomization	2b
		Positive: 5	CONTRA:	
		Uncertain: 3	Patients & blinding: small sample size (*n* = 131); patients not blinded; not placebo-controlled; high rate of Drop-Out because of non-compliance	
		Negative: 1	Methods: not all outcomes measured in numbers (e.g. psychological distress); diet of control group not further explained	
		Overall quality: Acceptable		
Lende [27]	RCT	SIGN	PRO: poweranalysis was conducted; structured explanation of randomization, structured explanation of Follow-Up	1b
		Positive: 5	CONTRA:	
		Uncertain: 1	Patients & blinding: high rate of Drop-Out; patients not blinded	
		Negative: 2	Methods: treatment (OP) individually different (different outcome possible); no numbers given about adverse events	
		Overall quality: Acceptable		
Lugtenberg [24] same study as DeGroot [23]	RCT	SIGN	PRO: detailed examination of side effects of fasting	2b
		Positive: 4	CONTRA:	
		Uncertain: 4	Patients & blinding: small sample size (*n *= 131); patients not blinded; not placebo-controlled; high rate of Drop-Out because of non-compliance	
		Negative: 1	Methods: ITT & PP-analysis do not agree (no explanation given); diet of control group not further explained	
		Overall quality: Acceptable		
Riedinger [25]	RCT	SIGN	PRO: structured explanation of statistical analysis; homogeneity in treatment (chemotherapy drugs)	2b
		Positive: 4	CONTRA:	
		Uncertain: 3	Patients & blinding: very small sample size (*n* = 24); only women; patients not blinded; not placebo-controlled	
		Negative: 2	Methods: study based on honesty of patients (compliance), no bloodwork was drawn during intervention	
		Overall quality: Acceptable		
Zorn [26]	Cross-over	SIGN	PRO: good comparison of the groups due to cross-over; bloodwork was drawn to measure compliance; authors discuss other studies with the same key question	2c
		Positive: 3	CONTRA:	
		Uncertain: 4	Patients & blinding: small sample size (*n* = 51); patients not blinded; not placebo-controlled	
		Negative: 2	Methods: double intervention with ketogenic diet; no randomization; QoL-data based on patient records; primary endpoint was not met	
		Overall quality:		
Dorff [29]	Cohort	SIGN	CONTRA:	2b
		Positive: 6	Patients & blinding: very small sample size (*n* = 13); patients not blinded; no control group	
		Uncertain: 2	Methods: study based on honesty of patients (compliance); different types of chemotherapy; randomization not specified; blood work was drawn at different times between the groups	
		Negative: 3		
		Overall quality: Acceptable		
Safdie [15]	CS	IHE	CONTRA:	4
		Positive: 5	Patients & blinding: very small sample size (*n* = 10); inclusion/exclusion criteria not defined; patients not comparable; data based on subjective questionnaire	
		Uncertain: 2		
		Negative: 2		
		Overall quality: Acceptable		

*IHE * Institute of Health Economics- Quality Appraisal Checklist for Case Series Studies, *SIGN* Scottish Intercollegiate Guidelines Network Methodology: Checklist 2: Randomised Controlled Trials

### Quality of life

Five of the included studies examined whether fasting before receiving chemotherapy, CT scan or surgery influenced the quality of life of the participants.

Bauersfeld et al. [16] conducted a prospective randomised cross-over trial including 50 female patients suffering from breast- and ovarian cancer. Patients went through a total of six cycles of chemotherapy. One group fasted for the first three cycles and ate a normocaloric diet for the second three cycles, while the other group performed this schedule the other way around. Fasting period was limited to 36 h before, and 24 h after, treatment. Between the cycles, patients were instructed to return to their usual diet. The results reported a significant increase in quality of life during the fasting chemotherapy cycles compared to the non-fasting cycles measured eight days after the chemotherapy treatment in one group (e.g. FACT-G scale: mean difference = 9.2; *p *value = 0.04) but not in the other group (mean difference = 2; *p* > 0.05), although the study was not powered to assess such an outcome. Due to the cross over nature of the study, however, the comparability is given. Different scales were used when measuring this outcome (Functional Assessment of Cancer Therapy-General FACT-G, Functional Assessment of Cancer Therapy-Fatigue FACT-F, Trial Outcome Index TOI) and the same significant trend was seen. However, reported results with relation to quality of life revealed missing in all tables and no statistical calculations were reported. Patients of the initial fasting group did not comply with the cross-over-design as they did not want to return to normal eating after the first three cycles. Lastly, the significant increase in quality of life has only been found in one of the groups and, therefore, was not replicated in the other group, thereby reducing the power of this conclusion even further. Stratification and testing of multiple outcomes add to those methodological limitations of this rather small study.

De Groot et al. [23] conducted an RCT among 131 female breast cancer patients, which was examined with regards to quality of life by Lugtenberg et al. in a second publication [24]. Patients in the fasting group followed a diet with a total duration of four days, starting with 1200 kcal on day 1 and moving to 200 kcal for day 2–4. The diet was obeyed three days prior to and on the day of each chemotherapy cycle. Patients underwent 6–8 cycles in total. Both authors did not find any significant differences in this study between the fasting group (IG) and the group consuming a regular diet (CG) (e.g. QoL EORTC QLQ-30 Global IG 97.5 vs. CG 80.5; *p* > 0.05); however, Lugtenberg et al. [24] reported that the patients who fasted had a changed illness perception and less worries in general (*p* < 0.05). Nonetheless, it must be considered, that patients tend to be especially mindful and observant when participating in an intervention study when compared to usual care. However, the intention-to-treat analysis does not concur with the conclusion presented. In fact, the high drop-out (33 patients out of 66 in the intervention group were not compliant), not only refutes this conclusion, but also lowers the numbers of evaluable participants and reduces the methodical strength. The high drop-out furthermore provides evidence questioning the feasibility of fasting in this vulnerable population. Lastly, no description of the dietary intake in the control group is provided, which can be subject to large intra- and interindividual variability and thus may also influence the outcome.

In another RCT, Riedinger et al. [25] included 24 female subjects suffering from gynaecological cancer. In this trial, one half of the patients followed a water-only diet 24 h before and 24 h after receiving chemotherapy in 3-week intervals for a minimum of six cycles. The other half maintained their regular normocaloric diet. Quality of life was measured according to the NCCN FACT FOSI-18 score. No significant difference between the two groups was found with regard to quality of life (*p* > 0.05). However, the authors report a tendency for increased quality of life in the fasting group when compared baseline between the intervention group (IG) and the control group (CG) (Arm A 58.38 vs. Arm B 57.1; *p* = 0.015). However, no data regarding the baseline in both groups are presented. Furthermore, the low drop-out rate (*n* = 1) is quite surprising when compared to the results from similar interventions. As in general no side effects of the short-term fasting regime were reported, it remains to be ascertained why the drop-out rate was so low. Interestingly, the subject dropped out due to social isolation. Lastly, compliance to the dietary regime was measured through subjective statements derived via telephone calls or in personal interviews, and no objective data such as serum samples were used to confirm compliance.

Zorn et al. [26] conducted a controlled cross-over study with the double intervention of fasting in combination with a ketogenic diet. The study included 51 female patients with gynaecological cancer. Patients were divided into four groups. They followed either a modified short-term fasting regime in which energy intake was limited to 400 kcal three days before and one day after chemotherapy. This regime was repeated for 2–3 cycles and subjects were instructed to return to their normal diet for the next 2–3 cycles. Group A and B performed this cycle in opposite directions. For Group C and D, a second intervention introduced a normocaloric ketogenic diet six days prior to the modified short-term fast. No significant difference in quality of life was found when comparing the fasting group with the control group, nor when comparing the fasting group with the ketogenic fasting group (*p* > 0.05). Serum samples were drawn to control the compliance of the patients. Similar to Bauersfeld et al. [16], Zorn et al. reported a high drop-out in spite of the fact that randomisation was done through selective allocation and not randomisation.

In the randomised controlled trial by Lende et al. [27] 80 female patients who were scheduled to undergo breast cancer surgery were included. The patients were instructed to fast according to the standard perioperative fasting protocol before surgery and provided access to free tap water. The control group received a different peri-operative protocol consisting of carbohydrate-based oral nutrition supplements (ONS 400 ml “pre-Op Nutricia” on the evening before, and on the day of, the operation). There were significantly more reports of mild and moderate pain seven days post-surgery in the fasting group when compared to the group who received ONS (post-hoc analysis; day 5 fasting 47% vs. carbohydrates 28%, *p* = 0.038; day 6 fasting 50% vs. carbohydrates 28%, *p* < 0.001).

### Chemotherapy-related toxicities

A randomised controlled pilot trial by de Groot et al. including 13 female patients with breast cancer was conducted in 2015 [28]. Patients in the intervention group fasted 24 h before and 24 h after treatment for a total of six cycles of chemotherapy. The results did not differ significantly from a larger study also published by Groot et al. in 2020 [23]. In this study, chemotherapy-related toxicities grade III/IV were investigated among 131 breast cancer patients. No significant differences were found with regard to chemotherapy-induced side effects grade I/II compared to the control group (fatigue, infection, mucositis, neuropathy, diarrhoea, dizziness, nausea, eye complaints, constipation) or grade III/IV (neutropenic fever, fatigue, infection) (e.g. fatigue IG 5 vs. CG 6; dizziness IG 3 vs. CG 3; *p* > 0.05). In fact, 43/65 participants in the intervention group (66%) reportedly disliked distinct components of the diet and were therefore non-compliant for up to half of the 6–8 chemotherapy cycles.

Riedinger et al. [25] conducted a controlled trial only allowing the consumption of water. Similar to de Groot et al. [23, 28], no significant differences in side effects grade III/IV (IG 0 vs. CG 2; *p* = 0.49), including anaemia grade 2 (IG 1 vs. GC 3; *p* = 0.73), thrombocytopenia grade 2 (IG 0 vs. CG 1; p not stated) or neutropenia grade 2/3 (IG 4 vs. CG 2/IG 0 vs CG 1; *p* not stated) could be ascertained between the intervention group and the control group.

In the cross-over trial by Zorn et al. [26], no significant differences in chemotherapy-related CTCAE grade I/II toxicities were found with exception of stomatitis. Stomatitis occurred significantly less during the fasting cycles vs. the normocaloric cycles [mean fasting cycles 3 (5.5%) vs. normocaloric cycles 15 (25.9%), *p* = 0.013]. Parameter estimates (PE) ± standard deviations (SD) represent the difference for each toxicity score points between cycles of normocaloric diet and those of fasting. Patient-reported toxicities measured one week after chemotherapy, identified a significant improvement in headaches [mean fasting cycles 1.18 (± 2.06) vs. normocaloric cycles 2.74 (± 4.3), parameter estimates − 1.8 (± 0.55) 95%CI − 2.89–(− 0.71), *p *value = 0.002], weakness [2.84 (± 4.25) vs. 7.11 (± 5.4), parameter estimates − 1.99 (± 0.87) 95%CI − 3.72–(− 0.26), *p *value = 0.024] and total toxicities [47.52 (± 33.21) vs. 56.36 (± 32.14), parameter estimates − 10.36 (± 4.44) 95% CI − 19.22–(− 1.5), *p *value = 0.023] between the fasted and non-fasted cycles. Nevertheless, the primary endpoint of this study was toxicities higher than grade III and this endpoint was not met as only one case of CTCAE grade III was documented. Because of this the results of secondary endpoints must be interpreted with caution. Methodological limitations such as the high drop-out rate and the randomisation through selective allocation restrict the clinical applicability. Furthermore, it is unclear, who performed the selective allocation assignment, which leads to an even higher risk of bias. The authors wanted to conduct a randomisation, but, due to the strict daily routines of the patients with their chemotherapy schedules, have not been able to. In fact, patients’ characteristics differed significantly between the groups; for example cancer severity, type of chemotherapy, etc.

Dorff et al. [29] conducted a cohort study including 17 female and three male participants. The main aim of this study was to assess if chemotherapy-related toxicities (fatigue, alopecia, nausea, vomiting, constipation, diarrhoea) were influenced by the duration of fasting. They compared fasting for 24 h before chemotherapy to fasting for 48 h before chemotherapy and to fasting for 48 h before and 24 h after chemotherapy (72 h in total). Significant differences were only found with regards to nausea and vomiting. The authors reported that the longer the fasting regime, the less of these problems occurred (nausea 24 h 100%, 48 h 86%, 72 h 43%, *p *value = 0.019; vomiting 24 h 83%, 48 h 43%, 72 h 0%, *p *value = 0.003). However, the reduced incidence of vomiting might be also due to the empty stomach. This study relied on patient-reported data and no randomisation was done.

A case series conducted by Safdie et al. [15] reported on the side effects of fasting among 10 patients with various types of cancer (4 breast, 1 oesophagus, 2 prostate, 1 lung, 1 uterine, and 1 ovarian cancer). A non-validated questionnaire which used items from the Common Terminology Criteria for Adverse Events of The National Cancer Institute for self-rating examined whether fasting is safe, feasible and assessed if side effects of chemotherapy can be reduced by fasting. The fasting duration and regimes varied greatly among the subjects, yet all patients reported less chemotherapy-induced toxicities during fasting. In fact, nausea, vomiting, diarrhoea, abdominal cramps, and mucositis were not reported when fasting, but were reported five out of six times when patients were not fasting. However, this is not surprising, considering the empty digestive tract. Fatigue and weakness were reported to be reduced, respectively, during fasting.

### Survival after surgery

Lende et al. [27] also examined the relapse-free breast cancer-specific survival among 61 post-surgical patients. Patients fasted before surgery while the control group received a carbohydrate-based ONS (400 ml pro-Op Nutricia) on the evening before, and on the day of, the operation. Relapse-free survival from baseline was significantly higher in the fasting group when compared to the non-fasting group [event at risk, 2/35 (94% survival) fasting vs. 6/26 (77% survival) non-fasting, *p *value = 0.049] and breast cancer-specific survival in ER + patients [event at risk, 0/29 (100% survival) fasting vs. 4/21 (81% survival) non-fasting, *p* value = 0.015]. No significant difference was detected for breast cancer-specific survival in oestrogen receptor negative patients. However, progesterone receptor status differed significantly between the groups due to the fact that more progesterone-receptor-positive patients were included in the fasting group [IG 28 (80%) vs. CG 13 (50%); *p *value = 0.014]. Additionally, fewer patients in the fasting group had high tumor proliferation [IG 3 (30%) vs. CG 7 (70%); *p* value = 0.038] although confounding factors such as the initial hormone status of the patient, or the outcome and extent of each individual surgery may have confounded the results. A high drop-out rate of 23% was reported. Blinding was not possible. Side effects in the fasting group such as nausea, mobilisation, dizziness, insecurity, and bleeding are briefly discussed but not supported by data and *p *values. No power calculation was reported with regard to the main outcomes. Therefore, it is not possible to conclude that the higher survival rate is due to the fasting regime.

### Side effects of fasting

Unwanted weight loss is known to affect clinical outcomes and have negative impacts on the prognosis of the cancer patient. Five out of nine studies included in this systematic review reported data related to the patients’ bodyweight and two RCTs reported no significant change in bodyweight. Firstly, Bauersfeld et al. [16] reported that the mean bodyweight in both groups did not change significantly from the beginning to the end of the trial (mean change of bodyweight group A − 0.7 kg, group B + 0.6 kg; *p* value > 0.3). Similarly, Riedinger et al. [25] reported an average decrease of − 1.1 kg in the fasting group from the start of the trial and an average decrease of − 0.84 kg in the control group without a significant difference between the groups (*p *value = 0.81). In the cohort study by Dorff et al. [29] only one patient failed to regain 25% of the lost weight during treatment and therefore was not eligible to fast again. Grade 1 weight loss, as defined by 5 to 10% from baseline was also observed among two patients. However, cancer patients who experienced a recent weight loss of 10% of their bodyweight were excluded from the study. In contrast three out of nine of the included studies did report a significant weight loss among their patients. In fact, Lugtenberg et al. [24] observed a decrease in body mass index (BMI) halfway through therapy (median decrease − 0.38 kg/m^2^; *p *value = 0.002) and at the end of the trial (median decrease − 0.33 kg/m2; *p *value = 0.026) in the fasting group, while the BMI in the control group increased compared to baseline (median 0.64 kg/m^2^; *p *value = 0.043). They also observed that, at the 6-month-follow-up, the fasting group was able to maintain their bodyweight, while the control group experienced further increase in their BMI (no *p *value reported). Furthermore, in the cross-over trial by Zorn et al. [26] a significant loss in mean fat mass (− 0.63 ± 0.23; *p* value = 0.008) and in bodyweight (− 0.84 ± 0.26; *p *value = 0.002) was seen during the fasting period. The lost bodyweight and fat mass were not regained and remained significantly reduced at the end of the trial (*p* value < 0.005). Safdie et al. [15] reported the loss of seven pounds (3.1 kg) and 20 pounds (9 kg) of bodyweight among two patients, but reported that these patients were able to regain lost weight afterwards.

In the case series presented by Safdie et al. [15], adverse side effects were not reported to be increased during the fasting chemotherapy cycles. In contrast, Bauersfeld et al. [16] reported incidents of headache, weakness and hunger. Zorn et al. [26] only observed discomfort. De Groot et al. [28] noted withdrawal from fasting after the third chemotherapy cycle because of pyrosis or recurrent febrile neutropenia; however, these side effects also occurred during the regular dietary cycles 4–6. Therefore De Groot et al. [28] concluded that these side effects were not related to the fasting intervention. In their larger study conducted in 2020 [23], the only observed side effect reported was the dislike of distinct components of the diet, which impacted compliance with the prescribed regime. Dorff et al. [29] reported fatigue as the most frequently reported side effect, followed by headaches, dizziness, hypoglycaemia, weight loss, hyponatremia and hypotension. Riedinger et al. [25] did not report any side effects.

## Discussion

### Summary of main results

The cross-over trial by Bauersfeld et al. [16] reported a significant increase in quality of life during the fasted chemotherapy cycles in one group of patients with breast- and ovarian cancer, but this was not replicated in the other group. De Groot et al. [23] and Lugtenberg et al. [24] did not find any significant changes in quality of life during fasted chemotherapy. Another trial by De Groot et al. [28] examined the chemotherapy-related toxicities but did not find any differences in the fasting group. A water-only diet was consumed by the patients in the RCT of Riediger et al. [25] during chemotherapy cycles. There was no significant difference between the groups in quality of life, nor in chemotherapy-related toxicities. A double intervention of fasting in combination with a ketogenic diet was done by Zorn et al. [26], but no significant difference in quality of life was found when comparing the fasting group with the control group, nor when comparing the fasting group with the ketogenic fasting group. There also was no significant difference in chemotherapy-related toxicities, except stomatitis which occurred significantly less during the fasting cycles. Lende et al. [27] found more reports of mild and moderate pain after surgery in the fasting group when compared to the group who received a carbohydrate-based oral nutrition supplement. Also, Lende et al. [27] reported in increase in relapse-free survival and breast cancer-specific survival in ER + patients in the fasting group. The cohort study by Dorff et al. [29] assessed whether chemotherapy-related toxicities are influenced by the duration of fasting. Nausea and vomiting occurred less the longer the fasting regime was. The case series by Safdie et al. [15] reported that fasting is safe, and patients reported subjectively fewer side effects.

All studies share similar risks of bias: Firstly, as blinding is not possible for such interventions, the risk of the Hawthorn effect should be addressed, particularly in studies which address quality of life as a main endpoint [16, 24, 25, 27] and those that rely on patient-reported data. In fact, Lugtenberg et al.’s RCT [24] showed, that fasting patients worried significantly less (*p* < 0.01). Furthermore, participants might underreport side effects. This can be explained with the Hawthorne effect. It concerns research participation, the consequent awareness of the patients of being studied, and therefore, possible impact on changing the behaviour of the patients [30]. In fact, more evidence exists confirming that the Hawthorne effect leads to underreporting among cancer patients in self-report questionnaires [31]. The risk of inherent bias is further compounded by the fact that with the exception of de Groot et al. [23] analysis was performed with per-protocol analysis. Furthermore, allocation bias occurs when patients are selectively assigned to the groups such as in the trial designed by Zorn et al. [26]. All these biases may strongly limit the reliability of the conclusion. Lastly, as fasting regimes for cancer patients have recently gained both media and scientific attention, the Hot Stuff bias may lead researchers to be less critical of their findings [32]. This is reflected in the fact that the conclusion reached by the authors exaggerates the value of their results and is neither supported by statistical power nor by the study design. Some patients also might have been fasting before the start of their study and therefore were already used to it. This probably increases the expectation bias, because the patients had intrinsic motivation to fast before the study.

### Quality of life

Among the nine studies heterogeneous results concerning quality of life are presented. While two studies [23, 26] found no difference, two [16, 25] found a significant increase in quality of life after chemotherapy treatment. Also, one study [27] found no significant increase in quality of life after surgery. The studies partially used different methods to assess quality of life, which can be found in the column “Endpoints” of Table 3. While Bauersfeld et al. [16] found a significant increase in quality of life when comparing the fasted cycles in one group to the non-fasted cycles of the same group, these results are limited by the study design. In fact, the sample size does not adequately power multiple hypothesis testing, and a partial failing of the cross-over occurred which rendered differences between the chemotherapy cycles unreliable. Riedinger et al. [25] found a significantly higher score in quality of life compared to baseline in the fasting group when compared to the regular diet group. But when comparing the mean of the two groups in quality of life after chemotherapy there was no significant difference. Again, this study was not powered to support conclusions about quality of life. The remaining RCTs did not find a significant difference in quality of life between the two groups. De Groot et al. [23] did not report any statistical calculations and only presented a graphical depiction making it difficult to replicate. When Lugtenberg et al. [24] reported on this same study in a separate published analysis, no significant improvement was shown. Lende et al. [27] reported an increase in pain after surgery among participants in the fasting group which could adversely affect quality of life. Finally, Zorn et al. [26] also stated there was no effect on quality of life that could be detected from fasting regimes.

### Chemotherapy-induced toxicities

Three RCTs [23, 25, 28] reported no significant difference with respect to chemotherapy-related toxicities. Relying on patient-reported outcomes, Zorn et al. [26] found a significant decrease in stomatitis one week after the treatment, and a significant decrease in headaches, weakness and frequency of side effects. Although the fasting patients postponed their scheduled chemotherapy treatments less often when compared to non-fasting patients, the high drop-out, lack of randomisation, missing details about the non-fasting phase, and the fact that the authors failed to meet the inclusion requirements for the pre-determined primary endpoint of the study, mean the reliability of these results is questionable.

Dorff et al. [29] described that the longer the fasting period lasted, the fewer side effects occurred (fatigue, alopecia, nausea, vomiting, constipation, diarrhoea). However, this study is one-armed without a control group and biological factors, such as the fact that some observations may be explained by the absence of food in the digestive tract, are not addressed.

Considering the limitations of the data presented, evidence to support the idea that fasting reduces side effects of chemotherapy is not given. Reported side effects of short-term fasting were mild; weakness, fatigue, and hypotension were listed as most common. While these side effects are reported as minor, they do indicate that fasting may do harm. This is particularly important when the data on unwanted weight loss and high drop-out rates are considered. Five of nine studies reported a high drop-out in the intervention group which most probably points to the high burden the fasting imposes on patients. This aspect was not adequately addressed in any of the studies.

Data regarding unwanted weight loss were mixed. This may be due to the fact that the majority of patients in the studies suffered from breast cancer (*n* = 258) compared to other cancers (*n* = 57). In contrast to patients with gastrointestinal cancer, this group of patients may more easily regain the weight lost during fasting days. This is even more the case as these patients often even tend to gain weight during their treatment. Yet, losing weight and regaining may not come up to a steady state as weight loss in case of cancer often is associated with loss of muscle mass [33–35]. In contrast, a quick gain of weight most probably is not due to building muscle mass.

### Limitations of this work

There are some limitations of this systematic review. First, only studies in English or German language were included. Therefore, the search for fasting in connection with cancer treatment can be expanded in future research. Second, only a small number of studies was included in this review, which can show a general trend, but larger numbers are needed to make it more transferable. Furthermore, due to the heterogeneity with regard to cancer types, interventions and endpoints of the included studies we could not conduct a meta-analysis.

## Conclusion

To summarise, the evidence does not support the conclusion that fasting has any beneficial effect on the quality of life of cancer patients during treatment. Also, evidence on fasting regimes reducing side effects and toxicities of chemotherapy is missing. In contrast, as the negative effects of unintentional weight loss are known to impact clinical outcomes severely, fasting is not indicated in this context and there is no reason for recommendation.

## Data Availability

All data generated or analysed during this study are included in this published article (and its supplementary information files).
